# Point-of-care LPS-induced TNF-α release testing for early sepsis risk stratification in the emergency department

**DOI:** 10.3389/fimmu.2026.1897997

**Published:** 2026-09-10

**Authors:** Berta Cisteró, Adrian Ceccato, Erika P. Plata-Menchaca, Veronica Monforte, Queralt Caus-Capdevila, Aina Areny-Balaguero, Elena Campaña-Duel, Marta Camprubí-Rimblas, Gemma Goma-Fernandez, Carla Guijarro, Patricia Salom, Monica Lopez, Juan Tajan, Tiago Teles De Castro, Vanessa Roman, Joan Vieyra, Judit Cubedo, Eduard Guerrero, Emilio Gené, Antonio Artigas, Enrique Hernández-Jiménez

**Affiliations:** 1 Emergency Department, Hospital Universitari Parc Taulí, Institut d’Investigació i Innovació Parc Taulí (I3PT-CERCA), Sabadell, Spain; 2 Medicine Department, Universitat Autònoma de Barcelona, Barcelona, Spain; 3 Critical Care Center, Hospital Universitari Parc Taulí, Institut d’Investigació i Innovació Parc Taulí (I3PT-CERCA), Department of Medicine, Universitat Autònoma de Barcelona, Sabadell, Spain; 4 Centro de Investigación Biomédica en Red en Enfermedades Respiratorias (CIBERES), Instituto de Salud Carlos III, Madrid, Spain; 5 Microbiology Section, Laboratory Medicine Department, Hospital Universitari Parc Taulí, Institut d’Investigació i Innovació Parc Taulí (I3PT-CERCA), Universitat Autònoma de Barcelona, Sabadell, Spain; 6 Department of Pharmacy and Pharmaceutical Technology and Physical Chemistry, University of Barcelona, Barcelona, Spain; 7 Loop Diagnostics, StartUB! - University of Barcelona, Barcelona, Spain

**Keywords:** bacteremia, emergency department, endotoxin tolerance, host immune response, LPS-TLR4-TNF-α pathway, sepsis, TNF-alpha

## Abstract

**Background and objective:**

Early changes in innate immune responsiveness precede overt organ dysfunction in sepsis. In emergency department (ED) patients with suspected infection and physiological deterioration, we estimated the prevalence of reduced LPS-induced TNF-α responsiveness, characterized the associated clinical outcomes, and assessed the diagnostic accuracy of a point-of-care LPS-induced TNF-α release assay (TARA) for Sepsis-3 adjudicated at 24 h.

**Methods:**

In this prospective observational cohort, adults with suspected infection and NEWS2 ≥3 were consecutively enrolled. Whole-blood samples collected at ED arrival (0 h) and 4 h later were stimulated *ex vivo* with LPS, and TNF-α release was measured using the TARA assay.

**Results:**

Of the 203 enrolled patients, 180 had valid paired TARA results and 142 fulfilled Sepsis-3 criteria within 24 h. Reduced LPS-induced TNF-α responsiveness, defined by a negative TARA result, was frequent (75%) and was associated with higher Sepsis-3 adjudication (83.0% vs. 66.7%). Using a parallel rule (0 or 4 h), negative TARA identified Sepsis-3 with 78.9% sensitivity (95% CI 71.4 to 84.8), 39.5% specificity (95% CI 25.6 to 55.3), 83.0% PPV, 33.3% NPV, LR+ of 1.30, LR- of 0.54, and 70.6% accuracy. Predictive values are prevalence-dependent and not transportable to unselected ED populations, whereas the likelihood ratios are. In prespecified low-risk subsets, sensitivity was 87.0% (95% CI 75.6 to 93.6) with NEWS2 <5 and 79.1% (70.8 to 85.6) with qSOFA ≤1. Using a composite endpoint (Sepsis-2/Sepsis-3), accuracy improved to 77.0% with maintained sensitivity (78.8%). In a parsimonious model adjusted for age and qSOFA, TARA remained independently associated with Sepsis-3 (adjusted OR 2.94, 95% CI 1.28 to 6.75; *p* = 0.011), with an optimism-corrected AUC of 0.729 after bootstrap internal validation.

**Conclusions:**

In ED patients with suspected infection and NEWS2 ≥3, point-of-care TARA identified a prevalent phenotype of reduced LPS-induced TNF-α responsiveness, with high sensitivity and low specificity for Sepsis-3, including in patients with low severity scores. This profile is consistent with an adjunctive rather than a stand-alone diagnostic role, requiring further confirmation in multicenter cohorts with broader ED case-mix.

## Introduction

Sepsis is a life-threatening organ dysfunction caused by a dysregulated host response to infection and remains a major cause of hospitalization and death worldwide (1–5). In the emergency department (ED), early recognition is challenging because presentations are often nonspecific and organ dysfunction may be absent or subtle at first contact. Diagnostic uncertainty can therefore delay timely antimicrobials and source control or, conversely, drive unnecessary broad-spectrum antibiotic exposure (6–8). Tools that improve early recognition and risk assessment at presentation are needed (8–10).

Current guidance states that sepsis is a clinical diagnosis that should not be established or excluded on the basis of a single biomarker or diagnostic test and identifies insufficient evidence to recommend novel and rapid host-response diagnostics, an evidence gap that the present study addresses (8). Current ED practice relies on clinical judgment, bedside scores (e.g., qSOFA, NEWS2), routine labs, and nonspecific inflammatory biomarkers (10), yet pathogen-based diagnostics are slow and do not capture the real-time host response (11). C-reactive protein (CRP) and procalcitonin (PCT) have limited sensitivity early in the course and variable specificity across heterogeneous ED populations (12–14), although recent ED data suggest that PCT can provide strong discrimination and incremental value when added to qSOFA-based assessment, highlighting the need to benchmark novel host-response assays against readily available comparators (15). As a result, early decisions are often made with incomplete information. These gaps have driven interest in host-response approaches, including gene-expression signatures, but bedside implementation remains limited by technical complexity (16). Sepsis is immunologically heterogeneous, and some patients develop early innate immune hyporesponsiveness consistent with an endotoxin-tolerance phenotype, marked by impaired cytokine production after lipopolysaccharide (LPS) stimulation (17–22). Monocyte HLA-DR and *ex vivo* LPS-stimulated cytokine release are established markers of sepsis-associated immune dysfunction (23, 24), yet ED use is constrained by standardization and operational barriers (23, 25).

The LPS-induced TNF-α release assay (TARA) is a rapid whole-blood functional immune point-of-care test compatible with ED workflows. Using a prespecified TNF-α threshold, it identifies blunted innate immune responsiveness and provides a pathway-specific functional signal early in suspected infection (26). The assay can be performed at the bedside by trained ED staff without specialized laboratory infrastructure. We aimed to estimate the prevalence of early sepsis-associated innate immune hyporesponsiveness, defined as reduced LPS-induced TNF-α responsiveness consistent with an endotoxin-tolerance phenotype, in ED patients with suspected infection and physiological deterioration, to characterize its associated clinical outcomes, and to assess the diagnostic performance of TARA for Sepsis-3 adjudicated within 24 h. We evaluated performance across infection foci, in clinically low-risk presentations, and versus alternative reference standards including a composite Sepsis-2/Sepsis-3 endpoint. In this single-center enriched ED cohort (NEWS2 ≥3), we generated preliminary estimates of the diagnostic performance of this functional immune point-of-care assay to inform multicenter validation.

## Methods

### Study design and setting

We conducted a prospective, single-center observational cohort study in the emergency department (ED) of a tertiary-care hospital. Consecutive adults (≥18 years) presenting with suspected acute infection at triage or during the ED stay and a National Early Warning Score 2 (NEWS2) ≥3 were eligible. NEWS2 ≥3 was used as a pragmatic clinical enrichment criterion to identify ED patients with suspected infection and early physiological deterioration in whom additional biological risk information could be clinically relevant. It was not intended as a diagnostic reference standard for sepsis or as a biological correlate of TARA. Only medical patients were included; patients evaluated for urgent or emergent surgical conditions, including trauma or acute surgical abdomen, were excluded. All patients received standard care according to the 2026 Surviving Sepsis Campaign guidelines (8). The study followed a prespecified protocol and was approved by the institutional review board.

### Participants and sampling

Whole-blood samples were obtained at ED arrival (0 h) and 4 h later. The exclusion criteria included pregnancy or lactation, known HIV or hepatitis C infection, chronic immunosuppressive therapy, prior solid-organ or hematopoietic stem cell transplantation, participation in another interventional study within 60 days, and inability to provide informed consent. Enrollment was consecutive during staffed research hours to minimize selection bias.

### LPS-induced TNF-α release assay

The LPS-induced TNF-α release assay (TARA; SeptiLoop^®^, Loop Diagnostics, Spain) is an *ex vivo* functional immune test performed on lithium–heparin whole blood (**Supplementary Figure 1**). The test can be performed by standard ED staff after brief training without specialized laboratory infrastructure, supporting its use in routine ED workflows. One milliliter of fresh whole blood was incubated with purified lipopolysaccharide on a dry heat block, and after 2.5 h of stimulation, TNF-α release was detected using a qualitative lateral-flow immunoassay. Results were available in under 3 h from sample collection. Based on prior analytical and functional assay characterization, a hyporesponsive result was predefined as a negative assay result, corresponding to stimulated TNF-α below the supported threshold range of 200 to 250 pg/mL. In accordance with STARD reporting recommendations, the direction of the index test is defined as follows: a negative assay result, that is, absence of a detectable test line, is the result treated throughout as indicative of sepsis, whereas a positive result denotes preserved LPS-induced TNF-α responsiveness (26).

Analytical validation of the device demonstrated a limit of detection of 250 pg/mL, 100% agreement under repeatability conditions assessed over 12 consecutive days by one operator at one site using one device lot, and 97.22% to 100% agreement under reproducibility conditions assessed across three testing sites, with two operators per site and two device lots. Full validation results, including interference, specimen stability, and hook-effect testing, have been reported separately (27). Because the readout is dichotomous and the analytical threshold lies close to the biological cutoff, misclassification is expected in samples with stimulated TNF-α near 200 to 250 pg/mL.

A negative result reflects pathway-specific blunted innate responsiveness (defined as reduced LPS-induced TNF-α responsiveness consistent with an endotoxin-tolerance phenotype) and does not by itself establish clinical or global immunosuppression or generalized immune dysfunction.

### Reference standard and outcomes

The primary reference standard was sepsis adjudication according to Sepsis-3 criteria within 24 h of ED presentation. Diagnosis and infection focus were independently adjudicated by at least two board-certified physicians blinded to the biological data. Secondary outcomes included infection focus, presence of bacteremia (defined as ≥1 positive blood culture within 24 h), organ dysfunction at ED arrival, ICU admission within 24 h, and in-hospital outcomes.

Because no universally accepted gold standard exists for the diagnosis of early sepsis, we additionally conducted a pre-specified sensitivity analysis using a composite reference standard defined as Sepsis-2 OR Sepsis-3 (28–30). Analyses using the composite Sepsis-2 OR Sepsis-3 reference standard were restricted to patients with complete data for Sepsis-2 criteria and adjudicated infection focus. Sepsis-2 was defined as suspected infection plus ≥2 systemic inflammatory response syndrome criteria (31). This composite standard was applied systematically to the entire cohort and to each infection focus (respiratory, urinary, abdominal, and others). The rationale was to capture both patients fulfilling Sepsis-2 criteria, who may represent early sepsis without overt organ dysfunction, and those meeting Sepsis-3 criteria, thereby reducing misclassification bias inherent to reliance on Sepsis-3 alone in early ED presentations.

Vital signs, bedside severity scores (qSOFA and NEWS2), routine laboratory parameters, and biomarkers (including C-reactive protein, procalcitonin, and lactate when available) were recorded at each time point. C-reactive protein was available in 141 of 180 patients (78.3%) and procalcitonin in 155 of 180 (86.1%). For subgroup and sensitivity analyses, qSOFA ≥2 and NEWS2 ≥5 were used as commonly applied clinical escalation thresholds. The diagnostic performance of the index test was evaluated under both reference standards, with recalculation of sensitivity, specificity, positive and negative predictive values, likelihood ratios, and overall accuracy. Subgroup analyses were prespecified but exploratory. No adjustment for multiple comparisons was performed; therefore, subgroup findings were interpreted according to the magnitude and precision of the estimates and were not considered confirmatory. A formal multiplicity correction was not applied because subgroup results are presented as descriptive estimates with confidence intervals rather than as hypothesis tests, all prespecified subgroups are reported irrespective of the direction of their results, and no comparative significance claims are made between subgroups.

### Data collection

Trained study coordinators collected data using standardized case report forms. Clinical variables, laboratory results, and management data during the ED stay and the first 24 h of hospitalization were extracted from electronic medical records. Blood sampling times were aligned with routine clinical workflows. All data were de-identified and stored in a secure database.

### Statistical analysis

Sample size was prespecified to include at least 105 septic (Sepsis-3) patients, providing a 95% CI half-width of 0.075 for sensitivity (*α* = 0.05) using conservative assumptions from prior development data. Continuous variables are reported as mean (SD) or median (IQR) and categorical variables as counts and percentages. Group comparisons used Student’s *t*-test or Mann–Whitney *U*-test for continuous variables and *χ*² or Fisher’s exact test for categorical variables. Diagnostic performance was assessed using sensitivity, specificity, positive and negative predictive values, likelihood ratio, and accuracy. The prespecified primary diagnostic strategy was a parallel rule, classifying TARA as a negative assay detected at either 0 or 4 h. Performance was evaluated overall and in predefined subgroups, including infection focus and triage low-risk presentations. The primary parsimonious multivariable logistic regression model included the TARA parallel result, age, and baseline qSOFA. Model discrimination was assessed using the area under the receiver operating characteristic curve, overall performance using the Brier score, and calibration using the calibration intercept and slope. Internal validation was performed with 2,000 nonparametric bootstrap resamples; optimism was estimated from the difference between model performance in each bootstrap sample and in the original dataset and was subtracted from the apparent estimates. A broader covariate-adjusted model was retained as an exploratory sensitivity analysis and presented as **Supplementary Materials** because the number of patients not fulfilling Sepsis-3 criteria was limited relative to the number of estimated parameters. Analyses were conducted using complete cases without imputation, and two-sided *p*-values <0.05 were considered statistically significant.

### Ethics

The study was conducted in accordance with the Declaration of Helsinki and applicable local regulations. Ethical approval was obtained from the institutional review board, and written informed consent was obtained from all participants or their legally authorized representatives (Septip: 2022/3004).

## Results

### Study population

**Figure 1:**
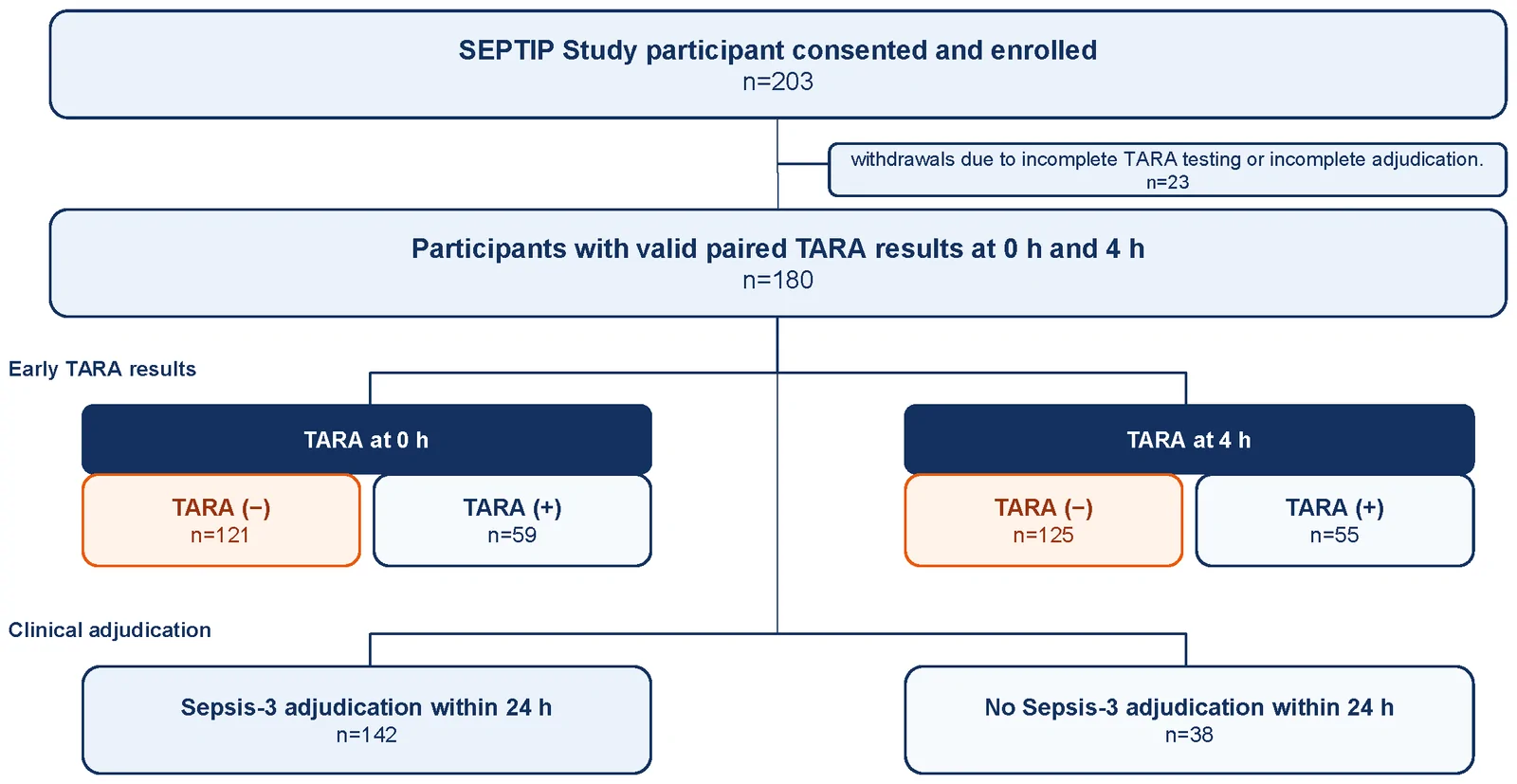
Overview of the study design and results for clinical adjudication. Consecutive adults presenting to the emergency department (ED) with suspected infection and a National Early Warning Score 2 (NEWS2) ≥3 were enrolled. Whole blood was collected at ED arrival (0 h) and 4 h later for *ex vivo* lipopolysaccharide (LPS) stimulation and measurement of tumor necrosis factor alpha (TNF-α) release using the point-of-care LPS-induced TNF-α release assay (TARA). Patients with valid paired TARA results at 0 and 4 h constituted the final analysis cohort. Sepsis status was independently adjudicated within the first 24 h after ED presentation according to Sepsis-3 criteria, defined as infection-associated organ dysfunction with an increase in Sequential Organ Failure Assessment (SOFA) score ≥2 points. The figure summarizes patient enrollment, exclusions, final analysis population, and classification according to Sepsis-3 adjudication.

A total of 203 adult patients with suspected infection and NEWS2 ≥3 at ED triage were enrolled. After adjudications and withdrawals, 180 patients (88.7%) had valid paired LPS-induced TNF-α release assay (TARA) results at 0 and 4 h and constituted the analysis cohort. Sepsis-3 criteria were fulfilled within the first 24 h in 142 patients (78.9%) (**Figure 1**).

### Baseline characteristics

Based on early TARA results at 0 and 4 h and on the combined 0–4 h parallel strategy (negative result at 0 and/or 4 h), patients having early immune hyporesponsiveness were classified as TARA-negative, whereas TARA-positive patients with preserved responses at both time points were classified as non-hyporesponsive. TARA classification was stable during the early assessment period in most patients, showing concordant results between 0 and 4 h (**Supplementary Table 1**).

The baseline demographic characteristics and most comorbidity profiles were comparable between groups, although dementia was more frequent among patients with preserved TARA responses. The distribution of infection focus differed between phenotypes, with respiratory infections being more frequent and urinary infections being less frequent among immune-hyporesponsive patients. Gram-negative microorganisms were less frequently identified among patients with reduced responsiveness (20.7% versus 40.0%), whereas the frequency of Gram-positive microorganisms was comparable between groups. This difference paralleled the distribution of infection focus since respiratory infections predominated among patients with reduced responsiveness, whereas urinary infections predominated among those with preserved responsiveness. Patients with early immune hyporesponsiveness more frequently fulfilled Sepsis-3 criteria and the composite Sepsis-2 or Sepsis-3 endpoint within 24 h. Hospital admission, ICU admission, ICU length of stay, time to ICU admission, in-hospital mortality, and secondary infection rates were similar between groups, noting that the study was neither designed nor powered for these outcomes and that the corresponding confidence intervals are wide, so the absence of a statistically significant difference should not be interpreted as evidence of absence of association, although time to first inpatient bed was longer among immune-hyporesponsive patients. Conventional severity scores and baseline biomarkers were broadly comparable across TARA phenotypes (**Table 1**).

**Table 1:** Baseline characteristics of all patients in total as well as all patients stratified by TARA result at 0–4 **(h)** .

Characteristic	Total (*N* = 180)	TARA 0 h (-) (*N* = 121)	TARA 4 h (-) (*N* = 125)	TARA 0–4 h (-) (*N* = 135)	TARA 0–4 h (+) (*N* = 45)	*p*-value
Baseline demographic						
Age, years	77 [63–85]	78 [63–85]	78 [63–85]	78 [63–85]	73 [63–83]	0.279
Female sex	60 (33.5%)	42 (35.0%)	50 (40.3%)	50 (37.3%)	10 (22.2%)	0.064
BMI, kg/m²	26.5 [23.1–29.8]	25.9 [23.1–29.4]	26.6 [23.1–29.7]	26.1 [23.1–29.5]	27.1 [23.8–31]	0.729
Myocardial infarction	23 (12.8%)	13 (10.7%)	15 (12.0%)	15 (11.1%)	8 (17.8%)	0.246
Heart failure	34 (18.9%)	21 (17.4%)	20 (16.0%)	23 (17.0%)	11 (24.4%)	0.272
Peripheral vascular disease	24 (13.3%)	16 (13.2%)	18 (14.4%)	20 (14.8%)	4 (8.9%)	0.311
Cerebrovascular disease	37 (20.6%)	22 (18.2%)	25 (20.0%)	25 (18.5%)	12 (26.7%)	0.241
Chronic pulmonary disease	51 (28.3%)	34 (28.1%)	38 (30.4%)	40 (29.6%)	11 (24.4%)	0.504
Dementia	45 (25.0%)	24 (19.8%)	26 (20.8%)	26 (19.3%)	19 (42.2%)	0.002
Liver disease	13 (7.2%)	7 (5.8%)	9 (7.2%)	9 (6.7%)	4 (8.9%)	0.740
Diabetes	47 (26.1%)	34 (28.1%)	34 (27.2%)	36 (26.7%)	11 (24.4%)	0.769
Chronic kidney disease	39 (21.7%)	26 (21.5%)	27 (21.6%)	29 (21.5%)	10 (22.2%)	0.917
Solid tumor malignancy	31 (17.2%)	21 (17.4%)	21 (16.8%)	24 (17.8%)	7 (15.6%)	0.732
Corticosteroid treatment	34 (20.0%)	25 (21.9%)	25 (21.4%)	27 (21.4%)	7 (15.9%)	0.431
Hematological disease	4 (2.2%)	3 (2.5%)	3 (2.4%)	3 (2.3%)	1 (2.2%)	1.000
HIV infection	1 (0.6%)	0 (0.0%)	1 (0.9%)	1 (0.8%)	0 (0.0%)	1.000
Connective tissue disease	1 (0.6%)	0 (0.0%)	1 (0.8%)	1 (0.7%)	0 (0.0%)	1.000
Source of infection						
Infection focus: respiratory	81 (45.8%)	62 (51.7%)	64 (51.6%)	67 (50.4%)	14 (31.8%)	0.032
Infection focus: urinary	52 (29.4%)	29 (24.2%)	32 (25.8%)	33 (24.8%)	19 (43.2%)	0.020
Infection focus: abdominal	27 (15.3%)	18 (15.0%)	19 (15.3%)	20 (15.0%)	7 (15.9%)	0.889
Infection focus: Others	17 (9.6%)	11 (9.2%)	9 (7.3%)	13 (9.8%)	4 (9.1%)	1.000
Gram-negative microorganism	46 (25.6%)	25 (20.7%)	25 (20.0%)	28 (20.7%)	18 (40.0%)	0.01
Gram-positive microorganism	16 (8.9%)	11 (9.1%)	9 (7.2%)	11 (8.1%)	5 (11.1%)	0.552
Clinical disposition and outcomes						
Sepsis-3 criteria	142 (78.9%)	100 (82.6%)	104 (83.2%)	112 (83.0%)	30 (66.7%)	0.020
Admitted to the hospital	159 (88.3%)	104 (86.0%)	106 (84.8%)	116 (85.9%)	43 (95.6%)	0.081
Admitted to ICU	31 (17.2%)	18 (14.9%)	19 (15.2%)	21 (15.6%)	10 (22.2%)	0.305
Time to first bed, h	15.2 [3–40]	25.5 [5.3–50]	21.8 [3.9–45.8]	24 [5.1–47.4]	9.4 [0–22.7]	0.005
ICU length of stay, days	5 [2.3–11.5]	5 [2.7–18.0]	3 [2–7]	5 [2–7]	5.7 [4.0–12.2]	0.319
Time to ICU, h	2.3 [-9–5.8]	2.3 [-9–5.4]	2.4 [-6.5–4.9]	2.4 [-8.3–5.5]	2 [-14–14.5]	0.962
In-hospital mortality	27 (15.2%)	19 (15.7%)	21 (16.9%)	22 (16.4%)	5 (11.4%)	0.417
Time from symptom onset to ED admission, h	61 [23.8–105.5]	59.4 [23.5–106.8]	51.8 [23.1–94.8]	57.4 [23.6–106.2]	66 [28.5–92.5]	0.949
Any secondary infection	12 (7.3%)	8 (7.1%)	9 (7.9%)	9 (7.3%)	3 (7.3%)	1.000
Conventional scores and biomarkers						
qSOFA score	1 [0–1]	1 [0–1]	1 [0–1]	1 [0–1]	1 [0–1]	0.604
NEWS2 score	5 [3–7]	5 [3–7]	5 [3–7]	5 [3–7]	6 [4–7]	0.082
SOFA score	3 [2–4]	3 [2–4]	3 [2–4]	3 [2–4]	2 [1–4]	0.170
APACHE score	11 [8–15]	11 [8.2–15]	11 [9–15]	11 [9–15]	10 [8–13]	0.288
Lactate, mg/dL	19.4 [12.3–29.8]	18.6 [12.4–30.8]	19.1 [12.4–30.1]	19.1 [12.4–29.9]	21.6 [12–29.4]	0.982
Procalcitonin (PCT), ng/mL	2.6 [0.6–8.2]	3.6 [0.5–9.0]	3.2 [0.6–8.2]	3.6 [0.5–9.0]	1.1 [0.8–7]	0.762
C-reactive protein (CRP), mg/dL	8.8 [3.3–21.8]	8.3 [3.0–23.0]	9.6 [3.1–25.3]	8.9 [2.8–23.6]	8.5 [4.8–16.1]	0.847
Leukocytes, ×10^9^/L	12.4 [8.0–17.5]	12.6 [7.3–17.5]	12.6 [7.5–18.2]	12.4 [7.3–17.2]	13 [10–17.3]	0.378
Neutrophils, %	86 [78–91]	87 [78–91]	86 [78–91]	86 [78–91]	84.5 [79–89]	0.667
Lymphocytes, %	7 [4–12]	7 [4–13.5]	7 [4.2–14]	7 [4–14]	7 [4–10]	0.528
Neutrophil/leukocyte ratio	0.9 [0.8–0.9]	0.9 [0.8–0.9]	0.9 [0.8–0.9]	0.9 [0.8–0.9]	0.8 [0.8–0.9]	0.667
Neutrophil/lymphocyte ratio	12.1 [6.6–21.6]	12.4 [5.9–21.8]	11.8 [5.2–19.4]	12.1 [5.2–21.5]	11.9 [7.8–21.6]	0.612

Continuous variables are reported as median [IQR]. Categorical variables are reported as n (%), with percentages calculated using the number of patients with available (nonmissing) data for each variable; percentages are column percentages unless otherwise specified. A negative TARA result indicates immune-hyporesponsiveness, defined as TNF-α below 200 -250 pg/mL after ex vivo LPS stimulation of whole blood. P-values compare TARA parallel 0 -4 h (−) versus TARA 0 -4 h (+) and were derived from the Mann -Whitney U-test for continuous variables and chi-square test or Fisher exact test for categorical variables. Missing data were not imputed, and no adjustment was made for multiple comparisons. Microbiology rows indicate a documented single classifiable Gram-negative or Gram-positive microorganism. One mixed case and no classifiable isolates were not assigned to either row. ED, emergency department.

### Diagnostic performance for Sepsis-3

When the reference standard was Sepsis-3 (**Supplementary Table 2**), using the prespecified parallel strategy (0 or 4 h), TARA demonstrated high sensitivity for Sepsis-3 adjudicated at 24 h, with moderate overall accuracy (**Table 2**). In comparison, qSOFA ≥2 showed high specificity but low sensitivity, whereas NEWS2 displayed intermediate performance profiles. Conventional biomarkers (CRP and PCT) showed lower diagnostic accuracy than TARA under the same strategy (**Supplementary Table 3**).

**Table 2:** Diagnostic performance of the TARA assay using a parallel rule (0 or 4 h) in the whole group, “low-risk” presentations, and by infection focus.

Group	*N*	Accuracy, % (95% CI)	Sensitivity, % (95% CI)	Specificity, % (95% CI)	PPV, % (95% CI)	NPV, % (95% CI)	LR+	LR−
TARA	180	70.6 (63.5–76.7)	78.9 (71.4–84.8)	39.5 (25.6–55.3)	83.0 (75.7–88.4)	33.3 (21.4–47.9)	1.30	0.54
Low-risk group								
qSOFA ≤1	153	69.3 (61.6–76.0)	79.1 (70.8–85.6)	39.5 (25.6–55.3)	79.8 (71.5–86.2)	38.5 (24.9–54.1)	1.31	0.53
NEWS2 <5	73	72.6 (61.4–81.5)	87.0 (75.6–93.6)	31.6 (15.4–54.0)	78.3 (66.4–86.9)	46.2 (23.2–70.9)	1.27	0.41
qSOFA ≤1 and NEWS2 <5	70	71.4 (59.9–80.7)	86.3 (74.3–93.2)	31.6 (15.4–54.0)	77.2 (64.8–86.2)	46.2 (23.2–70.9)	1.26	0.43
Infection focus								
Respiratory	81	67.9 (57.1–77.1)	82.5 (71.4–90.0)	16.7 (5.8–39.2)	77.6 (66.3–85.9)	21.4 (7.6–47.6)	0.99	1.05
Urinary	52	73.1 (59.7–83.2)	73.2 (58.1–84.3)	72.7 (43.4–90.3)	90.9 (76.4–96.9)	42.1 (23.1–63.7)	2.68	0.37
Abdominal	27	77.8 (59.2–89.4)	79.2 (59.5–90.8)	66.7 (20.8–93.9)	95.0 (76.4–99.1)	28.6 (8.2–64.1)	2.37	0.31
Bacteremia	58	69.0 (56.2–79.4)	74.5 (60.5–84.7)	45.5 (21.3–72.0)	85.4 (71.6–93.1)	29.4 (13.3–53.1)	1.37	0.56

The reference standard is Sepsis-3 adjudication. *N* indicates the number of patients in each subgroup. A negative TARA result, that is, absence of a detectable test line, denotes reduced LPS-induced TNF-α responsiveness and is the index test result treated as indicative of sepsis; a positive result denotes preserved responsiveness. Sensitivity, therefore, refers to the proportion of reference-positive patients with a negative TARA result and specificity to the proportion of reference-negative patients with a positive result. Performance measures are reported as accuracy, sensitivity, specificity, positive predictive value (PPV), negative predictive value (NPV), and positive and negative likelihood ratios (LR+ and LR-). Sensitivity and specificity 95% CIs were calculated using Wilson intervals. Subgroup estimates are exploratory and were not adjusted for multiple comparisons.

PPV, positive predictive value; NPV, negative predictive value; LR, likelihood ratio; qSOFA, quick Sequential Organ Failure Assessment; NEWS2, National Early Warning Score 2; TARA, LPS-induced TNF-α release assay.

### Performance in low-risk presentations and by infection focus

**Figure 2:**
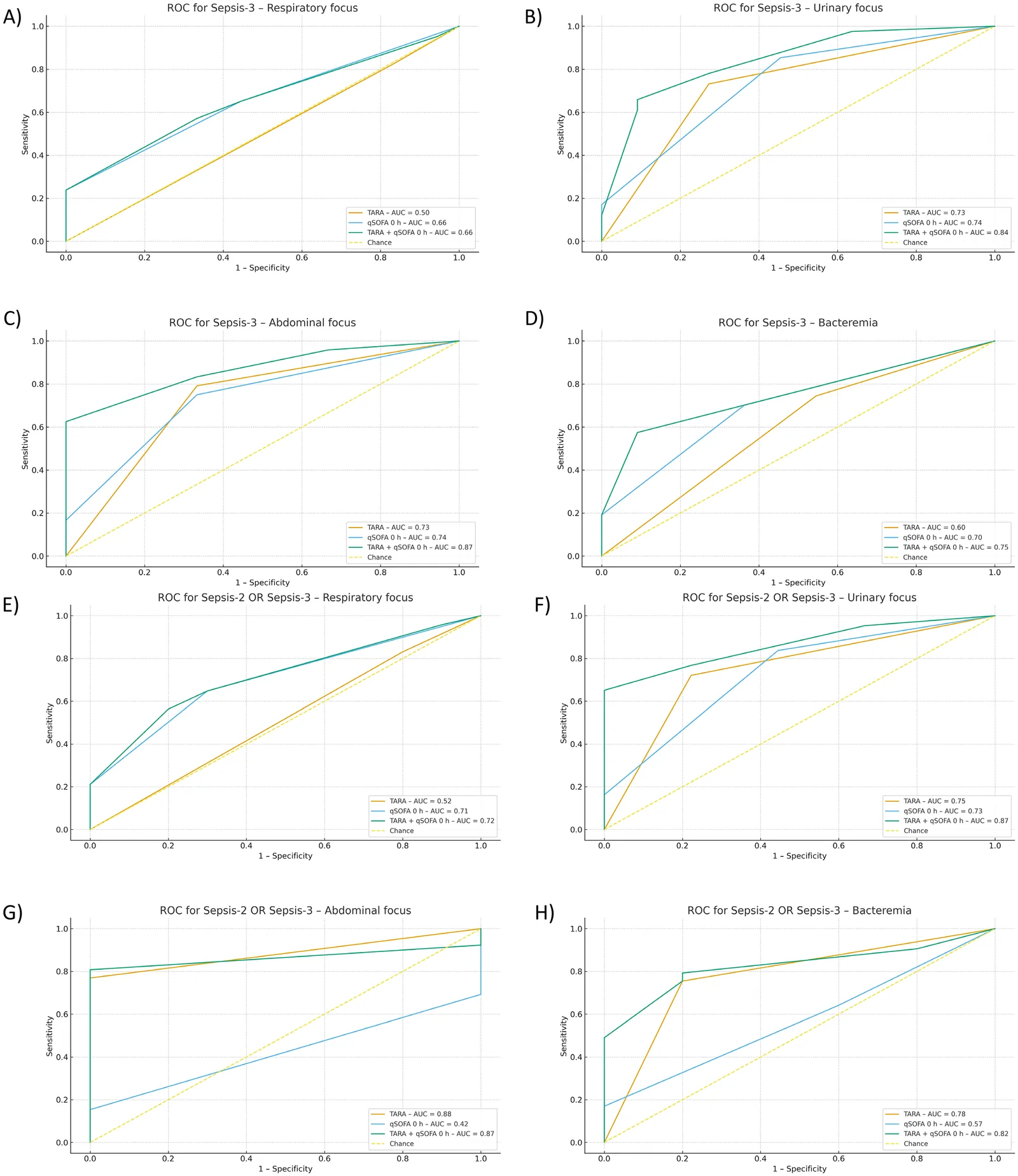
Discrimination of TARA and qSOFA for sepsis by infection focus. Receiver operating characteristic (ROC) curves for prediction of Sepsis-3 within 24 h are shown for TARA (parallel rule 0 or 4 h, binary), qSOFA at 0 h (continuous score 0–3), and the combined logistic regression model including both predictors. The diagonal dashed line denotes chance discrimination. The panels show prespecified subgroups by infection focus: **(A)** respiratory, **(B)** urinary, **(C)** abdominal, and **(D)** bacteremia. Receiver operating characteristic (ROC) curves for the prediction of Sepsis-2 and Sepsis-3 within 24 h are shown for TARA (parallel rule 0 or 4 h, binary), qSOFA at 0 h (continuous score 0–3), and the combined logistic regression model including both predictors. The diagonal dashed line denotes chance discrimination. The panels show prespecified subgroups by infection focus: **(E)** respiratory, **(F)** urinary, **(G)** abdominal, and **(H)** bacteremia. Area under the ROC curve (AUC) values for each model are reported within each panel. All subgroup AUCs are apparent, exploratory estimates obtained without internal validation or adjustment for multiple comparisons.

In predefined triage low-risk subsets (qSOFA ≤1 and/or NEWS2 <5), TARA retained high sensitivity with modest specificity, supporting its role as an adjunctive early case-finding tool when traditional scores suggested low severity. Diagnostic yield varied by infection focus. In respiratory infections, the largest single focus (*n* = 81), LR+ was 0.99 and LR- was 1.05, indicating essentially no discrimination, whereas performance estimates were numerically higher in urinary and abdominal infections and in bacteremia (**Table 2**). Estimates for the combination of TARA with qSOFA were numerically higher in urinary and abdominal sources (**Figure 2**).

### Secondary analysis using a composite reference standard

When the reference standard was broadened to a composite endpoint defined as Sepsis-2 OR Sepsis-3 (**Supplementary Table 4**), overall diagnostic accuracy improved while preserving high sensitivity (**Table 3**). Conventional biomarkers (CRP and PCT) showed lower diagnostic accuracy than TARA under the same strategy (**Supplementary Table 5**).

**Table 3:** Diagnostic performance of the TARA assay using a parallel rule (0 or 4 h) in the whole group, “low-risk” presentations, and by infection focus.

Group	*N*	Accuracy, % (95% CI)	Sensitivity, % (95% CI)	Specificity, % (95% CI)	PPV, % (95% CI)	NPV, % (95% CI)	LR+	LR−
TARA	174	77.0 (70.2–82.6)	78.8 (71.8–84.5)	61.1 (38.6–79.7)	94.6 (89.3–97.4)	25.0 (14.6–39.4)	2.03	0.35
Low-risk group								
qSOFA ≤1	147	76.9 (69.4–83.0)	79.1 (71.3–85.2)	61.1 (38.6–79.7)	93.6 (87.3–96.9)	28.9 (17.0–44.8)	2.03	0.34
NEWS2 <5	70	81.4 (70.8–88.8)	87.9 (77.1–94.0)	50.0 (25.4–74.6)	89.5 (78.9–95.1)	46.2 (23.2–70.9)	1.76	0.24
qSOFA ≤1 and NEWS2 <5	67	80.6 (69.6–88.3)	87.3 (76.0–93.7)	50.0 (25.4–74.6)	88.9 (77.8–94.8)	46.2 (23.2–70.9)	1.75	0.25
Infection focus								
Respiratory	77	79.2 (68.9–86.8)	83.1 (72.7–90.1)	33.3 (9.7–70.0)	93.7 (84.8–97.5)	14.3 (4.0–39.9)	1.25	0.51
Urinary	51	72.5 (59.1–82.9)	72.1 (57.3–83.3)	75.0 (40.9–92.9)	93.9 (80.4–98.3)	33.3 (16.3–56.3)	2.88	0.37
Abdominal	27	77.8 (59.2–89.4)	76.9 (57.9–89.0)	100.0 (20.7–100.0)	100.0 (83.9–100.0)	14.3 (2.6–51.3)	NA	0.23
Bacteremia	58	75.9 (63.5–85.0)	75.5 (62.4–85.1)	80.0 (37.6–96.4)	97.6 (87.4–99.6)	23.5 (9.6–47.3)	3.77	0.31

The reference standard is the composite Sepsis-2 or Sepsis-3 adjudication. *N* indicates the number of patients in each subgroup. A negative TARA result, that is, absence of a detectable test line, denotes reduced LPS-induced TNF-α responsiveness and is the index test result treated as indicative of sepsis; a positive result denotes preserved responsiveness. Sensitivity, therefore, refers to the proportion of reference-positive patients with a negative TARA result and specificity to the proportion of reference-negative patients with a positive result. Performance measures are reported as accuracy, sensitivity, specificity, positive predictive value (PPV), negative predictive value (NPV), and positive and negative likelihood ratios (LR+ and LR-). Sensitivity and specificity 95% CIs were calculated using Wilson intervals. Subgroup estimates are exploratory and were not adjusted for multiple comparisons. In the abdominal subgroup, only one patient was classified as reference-negative under the composite standard, so specificity was 100% with a very wide confidence interval, and LR+ was not estimable because the false-positive rate was zero; under the Sepsis-3 standard (**Table 2**), three patients were reference-negative in this subgroup. These estimates are not evidence of high specificity in abdominal infection and should be regarded as hypothesis-generating.

LR, likelihood ratio; NA, not estimable; NPV, negative predictive value; PPV, positive predictive value; qSOFA, quick Sequential Organ Failure Assessment; NEWS2, National Early Warning Score 2; TARA, LPS-induced TNF-α release assay.

This performance was maintained in low-risk subsets and across infection foci, with particularly numerically higher specificity and likelihood ratio estimates in urinary, bacteremia, and abdominal infections and in combination with qSOFA (**Table 3**, **Figure 2**).

### Multivariable modeling, internal validation

In the primary parsimonious multivariable model including the TARA parallel result, age, and baseline qSOFA, all 180 patients were included, giving approximately 13 events per variable for the limiting outcome category. TARA remained independently associated with Sepsis-3 diagnosis at 24 h (aOR 2.94, 95% CI 1.28 to 6.75; *p* = 0.011), together with qSOFA (aOR 3.71 per point, 95% CI 1.86 to 7.42; *p* < 0.001) and age (aOR 1.03 per year, 95% CI 1.00 to 1.05; *p* = 0.038) (**Table 4**). The model had an apparent AUC of 0.746; after internal validation using 2,000 bootstrap resamples, the optimism-corrected AUC was 0.729, the corrected Brier score was 0.152, and the corrected calibration slope was 0.921, indicating limited optimism and acceptable internal calibration. In a broader exploratory model additionally adjusting for sex, NEWS2, infection focus, diabetes, CKD, neoplasia, and corticosteroid use, TARA and qSOFA remained independently associated with Sepsis-3, whereas NEWS2 was not significant after adjustment (**Supplementary Table 6**). That model estimated 12 coefficients in addition to the intercept with only 34 patients without Sepsis-3, approximately three events per variable, and its higher apparent AUC of 0.81 illustrates the optimism expected at that ratio; it is therefore reported as exploratory. External validation of the primary model is planned in the multicenter study.

**Table 4:** Parsimonious multivariable logistic regression analysis of factors associated with sepsis-3 diagnosis.

Predictor	aOR	95% CI	*p*-value
SeptiLoop parallel (+ vs. −)*	2.94	1.28–6.75	0.011
Age (per year)	1.027	1.001–1.053	0.038
qSOFA at 0 h (per point)	3.71	1.86–7.42	<0.001

Model formula: Sepsis-3 ~ TARA parallel result (0–4 h) + age + qSOFA at 0 h. The clinically defined parsimonious model included the TARA parallel result, age, and qSOFA at presentation. A negative TARA result at 0 or 4 h, that is, absence of a detectable test line, denotes reduced LPS-induced TNF-α responsiveness and is the category modeled; preserved responsiveness is the reference. The analyses included all 180 patients with complete data for the three predictors, including 142 patients meeting Sepsis-3 criteria and 38 patients not meeting Sepsis-3 criteria. The results are reported as adjusted odds ratios (aORs), 95% confidence intervals (CIs), and *p*-values. The apparent model AUC was 0.746. After internal validation with 2,000 bootstrap resamples, the optimism-corrected AUC was 0.729, the corrected Brier score was 0.152, and the corrected calibration slope was 0.921.

AUC, area under the receiver operating characteristic curve; qSOFA, quick Sequential Organ Failure Assessment; TARA, LPS-induced TNF-α release assay.

## Discussion

In this prospective ED cohort enriched for suspected infection and early deterioration (NEWS2 ≥3), early *ex vivo* LPS-induced TNF-α hyporesponsiveness was common and clinically relevant. Using a prespecified threshold, 75% of the patients met the criteria within 4 h, and this phenotype was associated with increased odds of meeting Sepsis-3 at 24 h and a composite Sepsis-2/Sepsis-3 endpoint. These data suggest that many patients who later satisfy contemporary sepsis definitions already exhibit early, functionally downregulated innate immune responses consistent with host-response heterogeneity reported in molecular endotyping studies (32). Because reduced LPS-stimulated TNF-α reflects a pathway-specific functional signal rather than direct evidence of clinical immunosuppression, we use “innate immune hyporesponsiveness” as the preferred term.

TARA showed a sensitivity-oriented performance profile rather than strong stand-alone diagnostic discrimination (to rule-in or rule-out test for Sepsis-3). Applied to the pre-test probability of this cohort (78.9%), a negative result raises the probability of Sepsis-3 to approximately 83%, and a preserved response lowers it only to approximately 67%, so neither result crosses a threshold at which management would change on the test alone. Stand-alone performance is, nevertheless, not the criterion by which early sepsis tools are evaluated. Current guidance states that sepsis is a clinical diagnosis that should not be ruled in or ruled out using a single biomarker or diagnostic test (8), and the screening instruments currently recommended operate at comparable or lower accuracy: qSOFA was judged insufficiently sensitive for use as a single screening tool, with only 24% of infected patients qSOFA positive in the derivation study (33), and in a large comparative analysis NEWS2 showed the best absolute performance with a sensitivity of 73.1% alongside a positive predictive value of 6.5%, while qSOFA had the lowest sensitivity at 23.1% (8, 33). Modest likelihood ratios are therefore characteristic of this entire category of tools and reflect the absence of a biological gold standard for the syndrome. At the prespecified thresholds, TARA was more sensitive than CRP or PCT, whereas PCT was substantially more specific. CRP showed lower sensitivity and accuracy but greater specificity than TARA. These results indicate different operating characteristics rather than superiority of one biomarker and should be interpreted in view of the selected thresholds, missing biomarker measurements, and high Sepsis-3 prevalence. CRP was evaluated at 10 mg/L or above and PCT at 2 ng/mL or above; the latter threshold is oriented toward a higher likelihood of bacterial sepsis, whereas lower thresholds are more commonly applied in emergency department cohorts, so the comparison is threshold dependent. Presepsin and IL-6 have demonstrated potential diagnostic or incremental value in selected ED cohorts, with pooled sensitivity estimates of approximately 0.82 for presepsin and 0.78 for procalcitonin and wide between-study variation in thresholds and reference standards. However, these biomarkers quantify circulating concentrations, whereas TARA evaluates inducible cytokine-producing capacity after a standardized *ex vivo* stimulus. Neither presepsin nor IL-6 was measured in the present study, and no direct comparative conclusion can therefore be drawn. This pattern suggests that functional immune competence assessed by *ex vivo* stimulation provides information that is not redundant with routine bedside scores or early inflammatory biomarkers (34, 35). Mechanistically, reduced cytokine production following LPS stimulation reflects monocyte reprogramming and endotoxin tolerance, a central feature of sepsis-induced immunosuppression (17–22). The absence of differences in ICU admission, mortality, SOFA score, or lactate indicates that reduced LPS-induced TNF-α responsiveness should not be interpreted as a severity or prognostic marker in this cohort. The lower frequency of Gram-negative microorganisms among TARA-negative patients likewise argues against interpreting TARA as a surrogate marker of Gram-negative infection or direct endotoxin exposure. Four considerations bear on this observation. First, the comparison is confounded by infection focus since respiratory infections predominated among patients with reduced responsiveness and Gram-negative organisms are recovered less often from respiratory than from urinary sources. Second, tolerance-like reprogramming of innate immune cells is not specific to LPS exposure since heterologous and cross-tolerance are described after stimulation with non-LPS microbial ligands, including TLR2 agonists and Gram-positive cell wall components, and after viral infection and sterile inflammation. Third, a causative organism was documented in only a minority of patients, and a proportion received antibiotics before sampling, so microbiological classification is incomplete. Fourth, the assay measures current inducible capacity rather than exposure. The finding is therefore best described as a tolerance-like functional phenotype that may arise across heterogeneous infectious and inflammatory contexts.

From a diagnostic perspective, the assay functioned as a sensitivity-oriented early adjunct. Using Sepsis-3 as the reference standard, the prespecified parallel strategy (0 or 4 h) achieved high sensitivity (78.9%) with moderate accuracy (70.6%), whereas qSOFA ≥2 remained highly specific but identified only a minority of Sepsis-3 cases, resulting in lower overall accuracy. These trade-offs align with the intended roles of each approach: qSOFA as a high-specificity rule-in severity tool and functional immune testing as a means to detect early host-response dysregulation before overt organ dysfunction becomes clinically apparent (10). These comparisons should be interpreted with the direction of bias in mind: the reference standard is defined by organ dysfunction, and qSOFA shares components with the Sequential Organ Failure Assessment score, so comparisons involving qSOFA are subject to incorporation bias operating in its favor, whereas TARA is measured independently of the reference standard. Consistent with this role, TARA maintained high sensitivity in triage-negative subsets (qSOFA ≤1 and/or NEWS2 <5), supporting its potential role as a sensitivity-oriented adjunct subject to prospective clinical validation.

Diagnostic yield varied by infection focus. Estimates were numerically higher in urinary and abdominal infections, although the abdominal subgroup comprised only 27 patients and its specificity estimate was highly imprecise, so no claim of superior performance in that focus can be sustained. In respiratory presentations, where clinical and etiological heterogeneity is greatest, the assay did not discriminate against Sepsis-3. This likely reflects the marked clinical and etiological heterogeneity of respiratory syndromes at ED presentation, where viral infections, non-infectious exacerbations, early bacterial pneumonia, and mixed etiologies frequently overlap. In this setting, early functional immune dysregulation may precede, but not necessarily translate into, Sepsis-3-defined organ dysfunction within the first 24 h, resulting in lower apparent specificity. Consistent with this interpretation, performance improved when a composite Sepsis-2 or Sepsis-3 endpoint was applied, suggesting that part of the observed discordance reflects definitional timing and adjudication uncertainty rather than purely false-positive biology (36). More generally, when the reference standard is imperfect and the index test is intended to detect a host-response abnormality that may precede organ dysfunction, specificity is biased downward because patients classified as reference-negative may include early cases not yet meeting the Sepsis-3 criteria within the 24-h adjudication window. Consistent with this, specificity rose from 39.5% to 61.1%, LR+ from 1.30 to 2.03, LR- from 0.54 to 0.35, and accuracy from 70.6% to 77.0% under the composite standard. This interpretation cannot be settled with the present data and is presented as a hypothesis. Importantly, Sepsis-2-based criteria continue to be used in contemporary studies focused on early sepsis detection and host-response characterization (28, 29, 37), supporting their role as a valid sensitivity reference standard in this context.

From a clinical standpoint, the primary value of early sepsis tools in the emergency department lies in risk identification rather than maximal diagnostic accuracy at presentation. This is particularly relevant in respiratory infections, where physiological abnormalities captured by clinical scores such as qSOFA are frequent and often nonspecific. In this context, combining qSOFA with TARA integrates physiological severity with an orthogonal host-response signal, maintaining a sensitivity-oriented profile while adding biological interpretability and supporting early risk stratification and decision-making in heterogeneous presentations.

In the primary parsimonious multivariable analysis, the TARA parallel result remained independently associated with Sepsis-3 after adjustment for age and baseline qSOFA. Internal validation using 2,000 bootstrap resamples showed only limited optimism, with the apparent AUC decreasing from 0.746 to an optimism-corrected AUC of 0.729. The corrected calibration slope of 0.921 and Brier score of 0.152 indicated acceptable internal calibration for an exploratory model. These findings support a complementary association between functional immune responsiveness and clinical severity and provide the rationale for the planned multicenter validation designed to establish incremental clinical utility. The broader covariate-adjusted model showed greater optimism and imprecision because of the limited number of patients without Sepsis-3 relative to the number of parameters. Both analyses therefore remain exploratory and require confirmation in a larger independent multicenter cohort. Local triage practices may further influence test performance: in the study center, a NEWS2 threshold of ≥3 triggers sepsis-oriented management, whereas higher escalation thresholds are used elsewhere in several healthcare systems (38, 39). In settings with higher cutoffs, functional immune testing may offer greater incremental value by identifying early immune dysfunction in patients not yet flagged by severity-based scores.

Recent data further clarify the temporal positioning of functional immune assays relative to other immune biomarkers. While monocyte HLA-DR expression is a robust marker of sepsis-induced immunosuppression, it has limited utility during the very early phase of sepsis at ED presentation. In a large real-world cohort, mHLA-DR was not predictive within the first 48 h, whereas persistent downregulation from day 3 to 4 onward was strongly associated with mortality and ICU-acquired infections (24). An accompanying editorial emphasized that mHLA-DR is not suited for early ED stratification, where inflammatory mechanisms predominate (40). Experimental and clinical data suggest that preserved or increased TNF-α responsiveness precedes monocyte deactivation, supporting a temporal dissociation between early functional immune responsiveness captured by TARA and later phenotypic immunosuppression reflected by mHLA-DR downregulation.

Positioned within the current diagnostic landscape, TARA is not intended to replace pathogen-based diagnostics or high-specificity clinical rules but to provide a complementary host-response signal. In contrast to more complex host-response platforms requiring specialized laboratory infrastructure, this functional immune assay can be implemented at the bedside with minimal technical requirements. This operational simplicity supports its use in ED triage and facilitates potential implementation across diverse healthcare environments, including low- and middle-income settings, as part of multimodal early sepsis assessment strategies (16, 30, 41–44).

Several limitations warrant consideration. The single-center design and an enriched inclusion criterion (NEWS2 ≥3) increase sepsis prevalence and may lead to spectrum effects and unstable estimates for some metrics, particularly when cell counts are small (e.g., qSOFA≥2), which underscores the need for external validation in broader ED case-mix cohorts. This enrichment was intentional, as the study was designed to evaluate patients with suspected infection and early physiological deterioration, in whom additional test information could potentially influence early clinical decision-making and prompt rapid treatment adjustments. Nevertheless, because enrollment required NEWS2 ≥3, the cohort represents an enriched population rather than an unselected ED population with suspected infection. This design limits direct comparisons with NEWS2, and no claim of superiority over NEWS2 is made since enrollment conditional on NEWS2 ≥3 would render any such comparison circular and also prevents extrapolation to patients presenting with NEWS2 below 3. Because predictive values depend on prevalence, the positive predictive value of 83.0% and the negative predictive value of 33.3% observed here cannot be transferred to unselected populations; applying the observed likelihood ratios at a prevalence of approximately 30% would yield a positive predictive value of approximately 36% and a negative predictive value of approximately 81%. The likelihood ratios, rather than the predictive values, are the transportable quantities, although spectrum effects may also affect sensitivity and specificity themselves. A proportion of patients received antibiotics prior to sampling, not only reflecting real-world ED practice and potentially influencing host-response measurements but also supporting the pragmatic relevance of the cohort. The assay interrogates a single innate immune pathway related to LPS–TNF-α signaling and cannot capture the full spectrum of sepsis immunopathology, including viral-dominant syndromes or immune trajectories mediated through other axes (34). Finally, the dichotomous lateral-flow readout may contribute to misclassification near the decision threshold. Analytical validation of the device also identified negative interference from acetylsalicylic acid, ibuprofen, and etanercept at concentrations above usual therapeutic levels and positive interference from human anti-mouse antibodies. Because non-steroidal anti-inflammatory drug use is common among emergency department patients, this could contribute to misclassification in the direction of apparent hyporesponsiveness, although *ex vivo* testing in volunteers taking ibuprofen showed no interference. Future multicenter studies incorporating quantitative signal acquisition are needed to refine thresholds and evaluate pathway-embedded clinical impact (45, 46). Ferritin and monocyte HLA-DR were not prospectively collected in this ED cohort, precluding direct comparison with immune stratification approaches used in recent biomarker-guided immunotherapy trials such as ImmunoSep (47), which selected patients with established Sepsis-3 and pneumonia or bacteremia for precision immunotherapy using ferritin and mHLA-DR-based immune phenotyping. More broadly, recent advances in sepsis diagnosis and monitoring support the integration of complementary functional, phenotypic, molecular, and pathogen-based biomarkers within multimodal clinical assessment pathways (48).

In summary, early *ex vivo* TNF-α release testing identified a prevalent phenotype of reduced LPS-induced TNF-α responsiveness at ED presentation, which retained sensitivity in patients with low apparent clinical severity. The assay is best regarded as a sensitivity-oriented, pathway-specific investigational adjunct intended to complement, not replace, clinical assessment, routine biomarkers, and severity scores, and the present data do not establish clinical usefulness. These results support further multicenter evaluation of TARA-guided pathways integrating functional immune status with clinical severity and, where available, molecular host-response or rapid pathogen diagnostics to optimize early sepsis recognition and resource allocation.

## Data Availability

The raw data supporting the conclusions of this article will be made available by the authors, without undue reservation.
