# Supplementary material for: Point-of-care LPS-induced TNF-α release testing for early sepsis risk stratification in the emergency department
Source: Front Immunol. 2026 Sep 10;17:1897997. doi: 10.3389/fimmu.2026.1897997 (PMC13600838; doi:10.3389/fimmu.2026.1897997)
Supplement: Supplementary file 1 [file Table1.docx]

Supplemental Data File:

**Point-of-care LPS-induced TNF-α Release Testing for Early Sepsis Risk Stratification in the Emergency Department**

B Cistero^1^, A Ceccato^2,3,4^, EP Plata-Menchaca^6^, V Monforte^2^, Q. Caus-Capdevila^7^, A. Areny- Balaguero^2,3^, E Campaña-Duel^2,3^, M Camprubi^2,3^, G. Goma^2^, C. Guijarro^1^, P. Salom^1^, M Lopez^1^, J Tajan^1^, T Teles De Castro^1^, V Roman^5^, J Vieyra^5^, J Cubedo^5^, E Guerrero^5^, E Gene^1^, A. Artigas^2,3*^, E Hernandéz-Jiménez^5*^

1. Emergency Department, Hospital Universitari Parc Taulí, Institut d'Investigació i Innovació Parc Taulí (I3PT-CERCA), Sabadell, Spain.
2. Critical Care Center, Hospital Universitari Parc Taulí, Institut d'Investigació i Innovació Parc Taulí (I3PT-CERCA), Department of Medicine, Universitat Autònoma de Barcelona, Sabadell, Spain.
3. Centro de Investigación Biomédica en Red en Enfermedades Respiratorias (CIBERES), Instituto de Salud Carlos III, Madrid, Spain.
4. Microbiology Section, Laboratory Medicine Department, Hospital Universitari Parc Taulí, Institut d'Investigació i Innovació Parc Taulí (I3PT-CERCA), Universitat Autònoma de Barcelona, Sabadell, Spain
5. Loop Diagnostics, StartUB! - University of Barcelona, Barcelona, Spain
6. Medicine Department, Universitat Autònoma de Barcelona, Bellaterra, Barcelona, Spain.
7. Department of Pharmacy and Pharmaceutical Technology and Physical Chemistry, University of Barcelona, Spain.

Notes: A. Artigas and E. Hernández-Jiménez contributed equally as senior authors (co-senior authors)

*Corresponding to A. Artigas and E. Hernández-Jiménez (AArtigas@tauli.cat and enrique@loopdx.com)

**Supp. Fig. 1** | Components and point-of-care workflow of TARA assay (SeptiLoop®, novel CE marked device under IVDR class C).

**Supp Table 1** |Early trajectories of TARA-defined functional immune responsiveness between emergency department 0 h and 4 h. Patients with paired TARA measurements at emergency department arrival (0 h) and 4 h were included in this analysis. TARA (−) indicates early immune hyporesponsiveness, whereas TARA (+) indicates preserved functional immune responsiveness. Persistent TARA (−) and persistent TARA (+) were defined as concordant results at both time points, while fluctuating trajectories were defined as a change from TARA (−) to TARA (+) or from TARA (+) to TARA (−) between 0 h and 4 h. Data are reported as n (%), with percentages calculated using the total analysis cohort as the denominator. Directional changes in paired TARA classification were assessed using the exact McNemar test, and agreement between the two time points was quantified using Cohen’s κ.

| Panel A. Paired TARA classification at 0 h and 4 h | | | |
| --- | --- | --- | --- |
| TARA classification at 0 h | TARA (−) at 4 h | TARA (+) at 4 h | Total at 0 h |
| TARA (−) at 0 h | 111 | 10 | 121 |
| TARA (+) at 0 h | 14 | 45 | 59 |
| Total at 4 h | 125 | 55 | 180 |
|  |  |  |  |
| Panel B. TARA trajectories between 0 h and 4 h | | | |
| Persistent TARA (−) | TARA (−) at 0 h and 4 h | 111 | 61.7% |
| TARA (−) to TARA (+) | Change from TARA (−) at 0 h to TARA (+) at 4 h | 10 | 5.6% |
| TARA (+) to TARA (−) | Change from TARA (+) at 0 h to TARA (−) at 4 h | 14 | 7.8% |
| Persistent TARA (+) | TARA (+) at 0 h and 4 h | 45 | 25.0% |
| Stable TARA classification | Persistent TARA (−) or persistent TARA (+) | 156 | 86.7% |
| Fluctuating TARA classification | TARA (−) to TARA (+) or TARA (+) to TARA (−) | 24 | 13.3% |
|  |  |  |  |
| Panel C. Paired statistical assessment | | | |
| Statistic | **Value** | **Interpretation** |  |
| Exact McNemar test, p-value | 0.541 | No evidence of a directional change in classification between 0 h and 4 h |  |
| Cohen’s κ | 0.692 | Substantial agreement between the two assessments |  |
|  |  |  |  |
|  | | | |

**Supp Table 2** |Characteristics of Patients by Sepsis-3 Adjudication Result Values are median [IQR] for continuous variables and n (%) for categorical, with percentages calculated using the number of patients with available (nonmissing) data for each variable. p-values from Mann–Whitney U (continuous) and χ² or Fisher’s exact test (categorical). Abbreviations: ACE, angiotensin-converting enzyme; ARB, angiotensin receptor blocker; CNS, central nervous system.

| **Variable** | **Total (n=180)** | **Sepsis-3 (n=142)** | **No Sepsis-3 (n=38)** | **p-value** |
| --- | --- | --- | --- | --- |
| Age, years | 77.0 [63.0–85.0] | 78.0 [64.2–85.0] | 73.0 [57.2–81.0] | 0.060 |
| Female sex | 60 (33.3%) | 51 (35.9%) | 9 (23.7%) | 0.179 |
| Height, cm | 164.0 [157.0–170.0] | 165.0 [158.0–170.0] | 162.5 [156.2–171.2] | 0.641 |
| Weight, kg | 72.0 [62.0–81.0] | 71.5 [62.0–81.0] | 73.5 [66.0–82.5] | 0.571 |
| BMI, kg/m² | 26.5 [23.1–29.8] | 26.4 [22.9–29.4] | 26.5 [24.1–33.4] | 0.461 |
| Myocardial infarction (prior) | 23 (12.8%) | 19 (13.4%) | 4 (10.5%) | 0.788 |
| Heart failure | 34 (18.9%) | 29 (20.4%) | 5 (13.2%) | 0.360 |
| Dementia | 45 (25.0%) | 37 (26.1%) | 8 (21.1%) | 0.674 |
| Diabetes | 47 (26.1%) | 40 (28.2%) | 7 (18.4%) | 0.299 |
| Chronic kidney disease | 39 (21.7%) | 29 (20.4%) | 10 (26.3%) | 0.506 |
| Solid tumor malignancy | 31 (17.2%) | 23 (16.2%) | 8 (21.1%) | 0.475 |
| Hematologic disease | 4 (2.2%) | 4 (2.8%) | 0 (0.0%) | 0.580 |
| Liver disease | 13 (7.2%) | 9 (6.3%) | 4 (10.5%) | 0.478 |
| Beta-blockers | 39 (21.7%) | 29 (20.4%) | 10 (26.3%) | 0.506 |
| ACE inhibitors/ARBs | 45 (25.0%) | 34 (23.9%) | 11 (28.9%) | 0.532 |
| Calcium-channel blockers | 8 (4.4%) | 5 (3.5%) | 3 (7.9%) | 0.368 |
| Anticoagulants | 93 (51.7%) | 78 (54.9%) | 15 (39.5%) | 0.102 |
| Statins | 59 (32.8%) | 46 (32.4%) | 13 (34.2%) | 0.847 |
| Corticosteroids | 34 (18.9%) | 31 (21.8%) | 3 (7.9%) | 0.062 |
| Infection focus: respiratory | 81 (45.0%) | 63 (44.4%) | 18 (47.4%) | 0.854 |
| Infection focus: urinary | 52 (28.9%) | 41 (28.9%) | 11 (28.9%) | 1.000 |
| Infection focus: abdominal | 27 (15.0%) | 24 (16.9%) | 3 (7.9%) | 0.208 |
| Infection focus: skin and soft tissue | 6 (3.3%) | 5 (3.5%) | 1 (2.6%) | 1.000 |
| Infection focus: unknown | 6 (3.3%) | 2 (1.4%) | 4 (10.5%) | 0.019 |
| Infection focus: osteoarticular | 2 (1.1%) | 2 (1.4%) | 0 (0.0%) | 1.000 |
| Infection focus: CNS | 2 (1.1%) | 2 (1.4%) | 0 (0.0%) | 1.000 |
| Infection focus: endocarditis | 1 (0.6%) | 1 (0.7%) | 0 (0.0%) | 1.000 |

**Supp Table 3** | Diagnostic performance of the clinical scores (qSOFA, NEWS2) and CRP and PCT assay for Sepsis-3, at ED arrival (0-4 h). The reference standard is Sepsis-3 adjudication. For each strategy, a result was defined using a Parallel rule (0 h OR 4 h). Performance measures are reported as accuracy, sensitivity, specificity, positive predictive value (PPV), negative predictive value (NPV), and positive/negative likelihood ratios (LR+ and LR−). Sensitivity and specificity 95% CIs were calculated using Wilson intervals. LR+ is not estimable (NA) when specificity is 100%. CRP measurements were available for 141 of the 180 patients (78.3%), whereas PCT measurements were available for 155 patients (86.1%). Analyses of each biomarker were restricted to patients with available measurements and complete reference-standard data. Abbreviations: ED, emergency department; NPV, negative predictive value; PPV, positive predictive value; LR, likelihood ratio; qSOFA, quick Sequential Organ Failure Assessment; NEWS, National Early Warning Score; SIRS, Systemic Inflammatory Response Syndrome; TARA, LPS-induced TNF-α release assay.

| **Strategy** | **Accuracy** | **Sensitivity** | **Sensitivity 95% CI** | **Specificity** | **Specificity 95% CI** | **PPV** | **NPV** | **LR+** | **LR-** |
| --- | --- | --- | --- | --- | --- | --- | --- | --- | --- |
| qSOFA ≥2 | 40.6% | 24.6% | 18.3%–32.3% | 100.0% | 90.8%–100.0% | 100.0% | 26.2% | NA | 0.75 |
| NEWS ≥5 | 63.9% | 72.5% | 64.7%–79.2% | 31.6% | 19.1%–47.5% | 79.8% | 23.5% | 1.06 | 0.87 |
| CRP ≥10, mg/L | 49.4% | 41.5% | 33.8%–49.8% | 78.9% | 63.7%–88.9% | 88.1% | 26.5% | 1.97 | 0.74 |
| PCT ≥2, ng/mL | 67.1% | 63.5% | 54.9–71.6% | 80.6% | 64.0–91.8% | 92.6% | 36.7% | 3.27 | 0.45 |

**Supp Table 4** |Characteristics of Patients by Sepsis-2 or Sepsis-3 Adjudication Result Values are median [IQR] for continuous variables and n (%) for categorical with percentages calculated using the number of patients with available (nonmissing) data for each variable. p-values from Mann–Whitney U (continuous) and χ² or Fisher’s exact test (categorical). Abbreviations: ACE, angiotensin-converting enzyme; ARB, angiotensin receptor blocker; CNS, central nervous system.

| **Variable** | **Total (n=174)** | **Sepsis-3/2 (n=156)** | **No Sepsis-3/2 (n=18)** | **p-value** |
| --- | --- | --- | --- | --- |
| Age, years | 77.0 [63.0–85.0] | 78.0 [64.8–85.0] | 65.5 [51.5–80.5] | 0.019 |
| Female sex | 58 (33.3%) | 55 (35.3%) | 3 (16.7%) | 0.185 |
| Height, cm | 164.0 [157.0–170.0] | 164.0 [157.0–170.0] | 169.0 [159.0–171.0] | 0.585 |
| Weight, kg | 72.0 [62.0–81.0] | 71.0 [62.0–81.0] | 74.5 [69.8–85.5] | 0.324 |
| BMI, kg/m² | 26.6 [23.1–29.8] | 26.2 [22.9–29.7] | 27.0 [24.3–33.4] | 0.552 |
| Myocardial infarction (prior) | 23 (13.2%) | 21 (13.5%) | 2 (11.1%) | 1.000 |
| Heart failure | 34 (19.5%) | 31 (19.9%) | 3 (16.7%) | 1.000 |
| Dementia | 45 (25.9%) | 42 (26.9%) | 3 (16.7%) | 0.411 |
| Diabetes | 46 (26.4%) | 45 (28.8%) | 1 (5.6%) | 0.045 |
| Chronic kidney disease | 37 (21.3%) | 33 (21.2%) | 4 (22.2%) | 1.000 |
| Solid tumor malignancy | 29 (16.7%) | 25 (16.0%) | 4 (22.2%) | 0.507 |
| Hematologic disease | 4 (2.3%) | 4 (2.6%) | 0 (0.0%) | 1.000 |
| Liver disease | 12 (6.9%) | 11 (7.1%) | 1 (5.6%) | 1.000 |
| Beta-blockers | 39 (22.4%) | 33 (21.2%) | 6 (33.3%) | 0.243 |
| ACE inhibitors/ARBs | 43 (24.7%) | 36 (23.1%) | 7 (38.9%) | 0.155 |
| Calcium-channel blockers | 7 (4.0%) | 5 (3.2%) | 2 (11.1%) | 0.155 |
| Anticoagulants | 89 (51.1%) | 84 (53.8%) | 5 (27.8%) | 0.046 |
| Statins | 57 (32.8%) | 50 (32.1%) | 7 (38.9%) | 0.600 |
| Corticosteroids | 33 (19.0%) | 32 (20.5%) | 1 (5.6%) | 0.202 |
| Infection focus: respiratory | 78 (44.8%) | 69 (44.2%) | 9 (50.0%) | 0.803 |
| Infection focus: urinary | 51 (29.3%) | 47 (30.1%) | 4 (22.2%) | 0.592 |
| Infection focus: abdominal | 27 (15.5%) | 25 (16.0%) | 2 (11.1%) | 0.743 |
| Infection focus: skin and soft tissue | 5 (2.9%) | 5 (3.2%) | 0 (0.0%) | 1.000 |
| Infection focus: unknown | 5 (2.9%) | 3 (1.9%) | 2 (11.1%) | 0.084 |
| Infection focus: osteoarticular | 2 (1.1%) | 2 (1.3%) | 0 (0.0%) | 1.000 |
| Infection focus: CNS | 2 (1.1%) | 2 (1.3%) | 0 (0.0%) | 1.000 |
| Infection focus: endocarditis | 1 (0.6%) | 1 (0.6%) | 0 (0.0%) | 1.000 |

**Supp Table 5** | Diagnostic performance of the clinical scores (qSOFA, NEWS2) and CRP and PCT assay for Sepsis-3, at ED arrival (0-4 h). The reference standard is Sepsis-3 or Sepsis-2 adjudication. For each strategy, a result was defined using a Parallel rule (0 h OR 4 h). Performance measures are reported as accuracy, sensitivity, specificity, positive predictive value (PPV), negative predictive value (NPV), and positive/negative likelihood ratios (LR+ and LR−). Sensitivity and specificity 95% CIs were calculated using Wilson intervals. LR+ is not estimable (NA) when specificity is 100%. CRP measurements were available for 141 of the 180 patients (78.3%), whereas PCT measurements were available for 155 patients (86.1%). Analyses of each biomarker were restricted to patients with available measurements and complete reference-standard data. Abbreviations: ED, emergency department; NPV, negative predictive value; PPV, positive predictive value; LR, likelihood ratio; qSOFA, quick Sequential Organ Failure Assessment; NEWS, National Early Warning Score; SIRS, Systemic Inflammatory Response Syndrome; TARA, LPS-induced TNF-α release assay.

| **Strategy** | **Accuracy** | **Sensitivity** | **Sensitivity 95% CI** | **Specificity** | **Specificity 95% CI** | **PPV** | **NPV** | **LR+** | **LR-** |
| --- | --- | --- | --- | --- | --- | --- | --- | --- | --- |
| qSOFA ≥2 | 26.1% | 19.0% | 13.5–26.2% | 100.0% | 78.5–100.0% | 100.0% | 10.5% | NA | 0.81 |
| NEWS ≥5 | 63.6% | 64.2% | 56.2–71.5% | 57.1% | 32.6–78.6% | 94.1% | 13.1% | 1.50 | 0.63 |
| CRP ≥10, mg/L | 52.7% | 51.7% | 42.8–60.4% | 63.6% | 35.4–84.8% | 93.9% | 10.8% | 1.42 | 0.76 |
| PCT ≥2, ng/mL | 64.5% | 64.1% | 55.9–71.5% | 69.2% | 42.4–87.3% | 95.8% | 15.0% | 2.08 | 0.52 |

**Supp Table 6** |*.* Exploratory multivariable logistic regression analysis of factors associated with Sepsis-3 diagnosis. The prespecified model included TARA parallel result (0 h or 4 h), age, sex, qSOFA at 0 h, NEWS2 at 0 h, infection focus, diabetes, CKD, neoplasia and corticosteroids; analyses were restricted to complete cases. Results are reported as adjusted odds ratios (aORs) with 95% confidence intervals (CIs) and p values. A negative TARA result, that is, absence of a detectable test line, denotes reduced LPS-induced TNF-α responsiveness and is the category modelled; preserved responsiveness is the reference. Model discrimination is summarized by the area under the receiver operating characteristic curve (AUC), which is an apparent value estimated in the development data without internal validation. The model was fitted on 169 complete cases, with 34 patients not fulfilling Sepsis-3 criteria and 12 estimated coefficients, giving approximately 3 events per variable. Abbreviations: ED, emergency department; NEWS2, National Early Warning Score 2; qSOFA, quick Sequential Organ Failure Assessment; TARA, LPS-induced TNF-α release assay; CKD, chronic kidney disease.

| **Predictor** | **aOR** | **95% CI** | **p-value** |
| --- | --- | --- | --- |
| SeptiLoop Parallel (+ vs −) | **3.19** | 1.22–8.32 | **0.018** |
| Age (per year) | 1.03 | 1.00–1.06 | 0.055 |
| Female sex (vs male) | 1.11 | 0.42–2.94 | 0.827 |
| qSOFA (per point) | **2.55** | 1.04–6.24 | **0.040** |
| NEWS2 (per point) | 1.12 | 0.87–1.45 | 0.376 |
| Diabetes (yes vs no) | 1.35 | 0.47–3.82 | 0.577 |
| CKD (yes vs no) | 0.51 | 0.17–1.49 | 0.217 |
| Neoplasia (yes vs no) | 0.47 | 0.14–1.56 | 0.219 |
| Corticosteroids (yes vs no) | 3.23 | 0.80–12.97 | 0.099 |
| Focus: Urinary vs Respiratory | 1.18 | 0.41–3.38 | 0.760 |
| Focus: Abdominal vs Respiratory | 7.40 | 0.80–68.61 | 0.078 |
| Focus: Other vs Respiratory | 0.76 | 0.20–2.85 | 0.687 |

Formula (Sepsis-3 ~ TARA Parallel (0-4h) + Age + Sex + qSOFA(0h) + NEWS2(0h) + Infection focus + Diabetes + CKD + Neoplasia + Corticosteroids). **AUC = 0.81**
